# Nanostructured Luminescent Gratings for Sensorics

**DOI:** 10.3390/ma15228195

**Published:** 2022-11-18

**Authors:** Lyubov’ Borodina, Vladimir Borisov, Kirill Annas, Aliaksei Dubavik, Andrey Veniaminov, Anna Orlova

**Affiliations:** Center of Information Optical Technologies, ITMO University, 197101 St. Petersburg, Russia

**Keywords:** photopolymerization, diffusion, photoluminescence, quantum dots, monomer, polymer, holographic grating, laser scanning microscopy, photoluminescence decay, energy transfer

## Abstract

Two-dimensional holographic structures based on photopolymer compositions with luminescent nanoparticles, such as quantum dots, are promising candidates for multiresponsive luminescence sensors. However, their applicability may suffer from the incompatibility of the components, and hence aggregation of the nanoparticles. We showed that the replacement of an organic shell at the CdSe/ZnS quantum dots’ surface with monomer molecules of the photopolymerizable medium achieved full compatibility with the surrounding medium. The effect was demonstrated by luminescence spectroscopy, and steady-state and time-resolved luminescent laser scanning microscopy. We observed the complete spectral independence of local photoluminescence decay, thus proving the absence of even nanoscale aggregation, either in the liquid composition or in the nodes and antinodes of the grating. Therefore, nanostructured luminescent photopolymer gratings with monomer-covered quantum dots can act as hybrid diffractive–luminescent sensor elements.

## 1. Introduction

Volumetric photopolymer composite recording media for holography are widely used to create new holographic optical elements, photonic crystals, and sensors [1,2,3]. One of the most promising approaches for achievement of a high refractive index modulation that is needed for high diffraction efficiency or information capacity is based on photoinduced mass transfer, namely the interdiffusion of nanoparticles (referred to as neutral component) and monomers caused by spatially modulated photopolymerization [4,5].

Semiconductor colloidal quantum dots (QDs) have not only been extensively studied and used in biomedical imaging, theranostics, light conversion, and sensoric applications [6,7,8] because of their fascinating luminescent properties but have also become attractive candidates for the role of the neutral component in liquid photopolymerizable light-sensitive media for holography because the refractive index of QDs significantly exceeds the indices of other components. Therefore, volumetric structures with high refractive index modulation, and hence diffraction efficiency [9,10], can be obtained by holographic recordings in photopolymer materials with semiconductor nanocrystals. Amplified emission was demonstrated in distributed feedback structures combining luminescence and diffraction properties [11].

However, for the successful use of colloid QDs in light-sensitive materials for holography, it is vital to ensure their perfect compatibility with the components of the medium and avoid their aggregation, which would significantly deteriorate the optical and luminescent properties of recorded structures. The compatibility is controlled by stabilizer molecules located on the surface of the QDs, which also affect the QDs’ spectral properties [12,13].

In this study, we present an approach to stabilizing QDs in a photopolymerizable medium by replacing the original stabilizer with monomer molecules identical to those polymerized in the course of holographic recording.

Modern laser scanning microscopy, e.g., micro-Raman analysis, provides powerful tools for the visualization of holographic structures [5]. The present study of holograms with QDs was performed using laser scanning luminescence microscopy, which made it possible to acquire volumetric images of holograms, measure the local photoluminescence (PL) spectra, and study the diffusion of QDs by introducing the spatial inhomogeneity of their PL and observing its intensity profile.

## 2. Materials and Methods

We synthesized CdSe/ZnS semiconductor QDs originally stabilized by trioctylphosphine oxide (TOPO) shells, following the protocol described in [14,15]. The surface stabilizer was afterwards replaced as described in Section 3.

The monomer 2-carboxyethyl acrylate was purchased from Aldrich (Product No. 552348, CAS 24615-84-7. Chloroform, toluene, and isopropyl alcohol were purchased from Lenreaktiv, St. Petersburg, Russia. Bis-(2,6-difluoro-3- (1-hydropyrrol-1-yl) phenyl) titanocene (Irgacure 784, CAS 125051-32-3) was used as a photopolymerization initiator, as in ref. [16]. The chemicals were used without further purification.

The replacement of the original TOPO stabilizer was carried out as follows: a colloidal solution of QDs in toluene was washed and precipitated with isopropyl alcohol in a 1:1 ratio, then centrifuged for 3 min at 12,000 rpm. After precipitation, the supernatant liquid was removed from the solution, and the precipitate was dissolved in chloroform. Thereafter, the QD solution was again precipitated in the same way, and the precipitate was redissolved in 2-carboxyethyl acrylate and left in a shaker for 24 h for continuous stirring at 800 rpm.

The infrared spectra of the QDs were measured using a Tensor 27 FTIR spectrometer (Bruker Optik GmbH, Ettlingen, Germany) in TIR mode.

The average hydrodynamic size of the QDs was determined by the dynamic light scattering method using a Zetasizer Nano ZS analyzer (Malvern Panalytical, Worcestershire, UK).

The absorption spectra were recorded using an UV 3600 spectrophotometer (Shimadzu, Kyoto, Japan). The PL spectra were recorded using a Cary Eclipse luminescence spectrophotometer (Varian, Mulgrave, Australia).

Steady-state PL images and local spectra were recorded using an LSM 710 confocal microscope (Carl Zeiss Microimaging, Munich, Germany) based on a Zeiss Axio Imager Z1 upright stand and controlled with the ZEN 12 software package. A Zeiss 50 × 0.95 objective lens, a 63 × 0.75 Plan-Neofluar objective lens with adjustable spherical aberration correction, and a 405 nm diode laser were chosen for optimal microscopic image quality.

The PL decay kinetics of a holographic grating with QDs were measured using a MicroTime 100 laser scanning microscope (PicoQuant, Berlin, Germany) controlled by SymPhoTime software. The light source was an LDH-PC-405B pulsed laser (PicoQuant) with a pulse duration of 20 ps and a wavelength of 409 nm. To enable spectral selection of the detected radiation in the range of 430–780 nm, a tunable continuous filter monochromator b (Carl Zeiss, Oberkochen, Germany) with a 10 nm bandwidth was included in front of the photodetector.

## 3. Results and Discussion

### 3.1. Stabilization of the Surface of Quantum Dots by Monomer Molecules

To ensure the best possible compatibility of the QDs intended to be neutral particles in a composition for holographic recording, we decided to stabilize their surface with the same molecules of 2-carboxyethyl acrylate monomer that surrounded the QDs in the recording material and underwent photopolymerization in the course of holographic recording.

**Figure 1:**
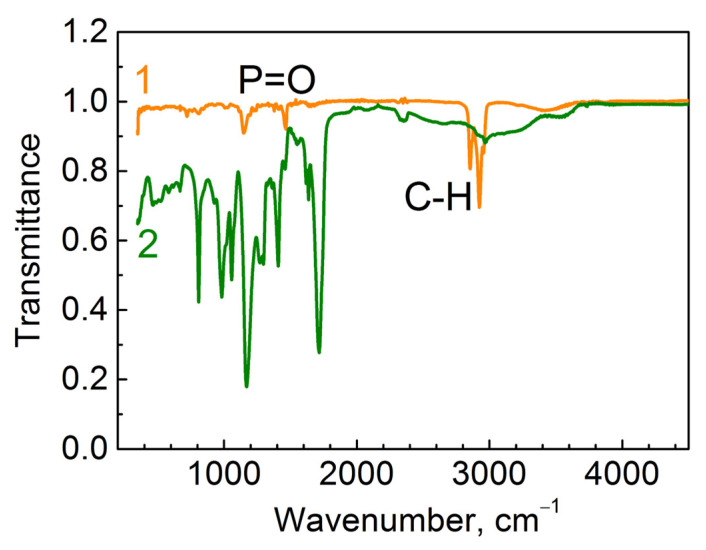
FTIR spectra of a layer of the synthesized quantum dots stabilized by trioctylphosphine oxide (TOPO) (1) molecules, and the quantum dots after replacement of the stabilizer with 2-carboxyethyl acrylate monomer molecules (2).

To confirm the complete replacement of TOPO on the surface of the nanocrystals by 2-carboxyethyl acrylate molecules, the FTIR spectra (Figure 1) of a layer of as-synthesized QDs stabilized by TOPO molecules (1) and QDs that underwent the stabilizer replacement procedure (2) were measured with a Tensor 27 spectrometer.

The Spectrum 1 in Figure 1 contains vibrations between 2800 cm^−1^ and 3000 cm^−1^, corresponding to the C–H band, and a characteristic peak at about 1467 cm^−1^, which refer to the valence vibrational band P=O [17]. Spectrum 1 indicates the presence of the initial stabilizer TOPO on the surface of the QDs. Spectrum 2 contains several bands in the range of 800–1700 cm^−1^ and a band at 2990 cm^−1^, which indicate 2-carboxyethylacrylate [18]. The absence of bands related to the TOPO ligand in the infrared spectrum (Spectrum 2) shows the complete replacement of TOPO by the monomer molecules.

**Figure 2:**
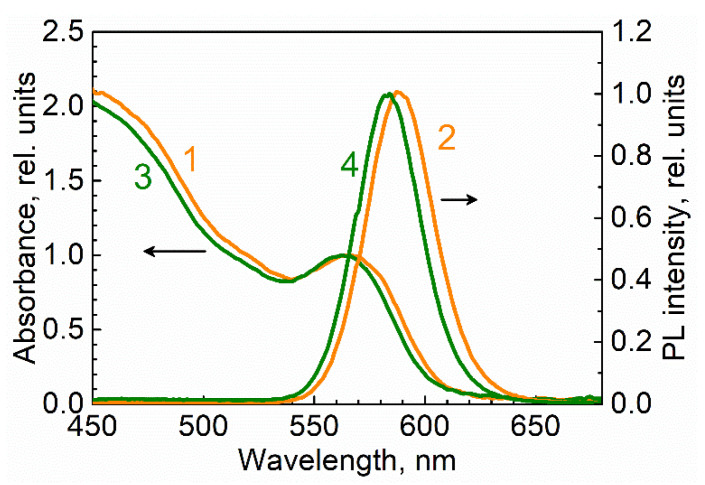
Absorption (1, 3) and normalized PL (2, 4) spectra of QD covered (stabilized) by TOPO in chloroform (1, 2) covered by and dissolved in 2-carboxyethyl acrylate (3, 4).

Figure 2 demonstrates the absorption (1, 3) and PL (2, 4) spectra of CdSe/ZnS QDs before (1, 2) and after (3, 4) replacement of the stabilizer.

According to the exciton absorption maximum at 565 nm, the average CdSe core diameter was calculated by Peng’s formula [19] as 3.5 nm, and the extinction coefficient at the exciton absorption maximum was calculated as 1.25·10^5^ M^−1^ cm^−1^. The maximum of the PL band of CdSe/ZnS QDs stabilized by the TOPO molecules was at a wavelength of 590 nm. Decorating the QDs’ surface with the monomer molecules led to the shift in the exciton absorption and PL maxima by a few nanometers to higher energies, similar to that observed in [14] resulting from exchanging TOPO with allylamine.

**Figure 3:**
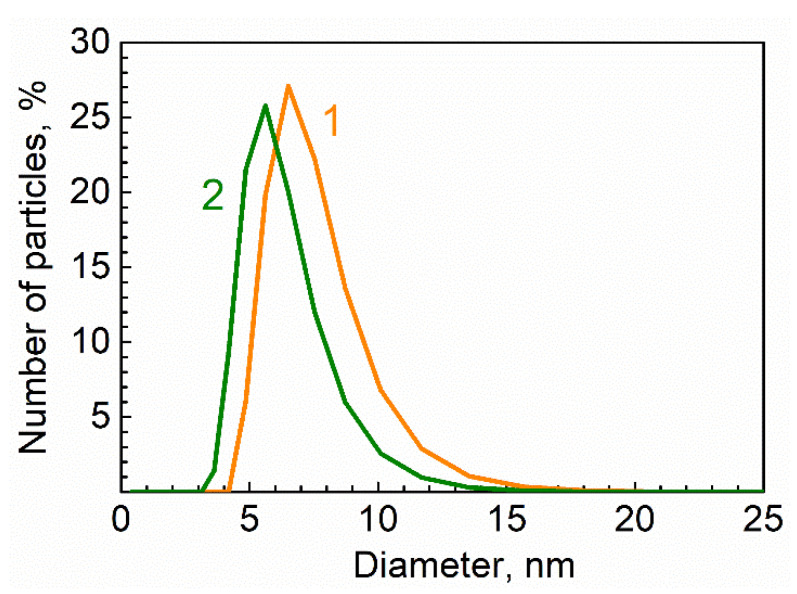
Size distribution of CdSe/ZnS quantum dots stabilized by TOPO (1) and 2-carboxyethyl acrylate (2) molecules.

Figure 3 shows the size distributions of the QDs stabilized by TOPO molecules (1) and 2-carboxyethyl acrylate monomer molecules (2), as measured by dynamic light scattering [20]. The hydrodynamic diameters of CdSe/ZnS QDs were determined as 7 nm and 6 nm for TOPO- and the monomer-stabilized QDs, respectively.

Therefore, the replacement of the QDs’ surface stabilizer did not cause significant changes in the hydrodynamic size of the QDs.

### 3.2. Composition of the Photopolymers 

The photosensitive composition used for holographic recording comprised the 2-carboxyethyl acrylate as a photopolymerizable monomer, Irgacure 784 as a photopolymerization initiator, and a 2-carboxyethyl acrylate-stabilized CdSe/ZnS QDs as the neutral component.

The components of the recording composition were combined in the following way: 1 mg of the photopolymerization initiator per specimen was added to 100 µL of the monomer-stabilized CdSe/ZnS QDs 10^−5^ M solution in the same 2-carboxyethyl acrylate monomer, then the composition was continuously stirred on a shaker for 24 h at a speed of 600 rpm for uniform distribution of the photopolymerization initiator throughout the volume of the monomeric composition. After that, a microliter droplet of the composition was placed and sealed between a slide and a cover slip, and a holographic grating was recorded by imprinting an interference pattern into it, as described below. Starting from when the initiator was added, the composition was handled and stored in the dark or, when visual control was necessary, under weak red lighting.

### 3.3. Holographic Recording

**Figure A1:**
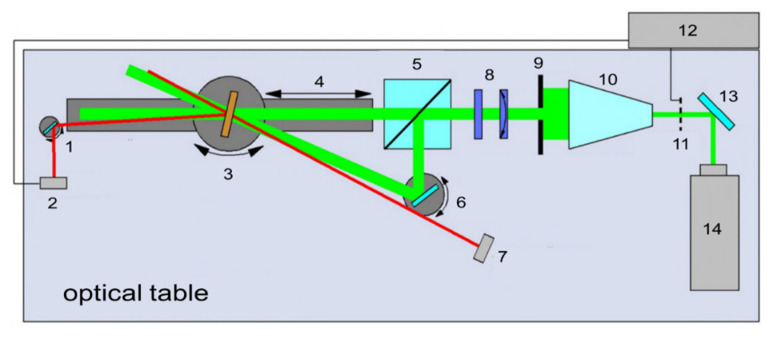
Scheme of the optical setup used for recording holographic gratings and monitoring the diffraction efficiency: 1—mirror mounted on a rotatable table, 2—power meter, 3—rotatable table with a specimen, 4—linear stage, 5—cubic beam splitter, 6—another mirror mounted on a rotatable table, 7—635 nm diode laser, 8—polarizing attenuator, 9—diaphragm, 10—telescopic beam expander, 11—shutter, 12—computer, 13—mirror, 14—532 nm DPSS laser.

Holographic recording was carried out using a diode-pumped solid-state continuous-wave Nd:YAG DPSS laser with frequency doubling (radiation wavelength 532 nm, output power 100 mW) in a symmetrical transmission holographic scheme, with the angle between two TE-polarized recording beams being 30°, as depicted in Appendix A Figure A1. The experimental setup was mounted on an antivibration-isolated optical table and was shielded against ambient dark red light and moving air.

To minimize the nonuniformity of the light beam’s cross-section, the laser beam passed through a telescopic beam expander with spatial filtering and a magnification of 100x. Through use of an iris diaphragm, the most uniform central part of the light beam (1 cm in diameter) was isolated with a relative decrease in intensity at the edges of <1%. The radiation power density in each arm of the interferometer was 4.8 mW/cm^2^, the exposure duration was 300 s, and the exposure energy density (dose) was 2.8 J/cm^2^. In the course of holographic recording, the diffraction efficiency was monitored with a diode laser emitting at 635 nm.

As a result of the holographic recording, a holographic grating with a period of 2.5 μm was obtained, the PL properties of which were then examined by means of steady-state and time-resolved laser scanning luminescence microscopy.

### 3.4. Steady-State Laser Scanning Microscopy of the QD Grating

**Figure 4:**
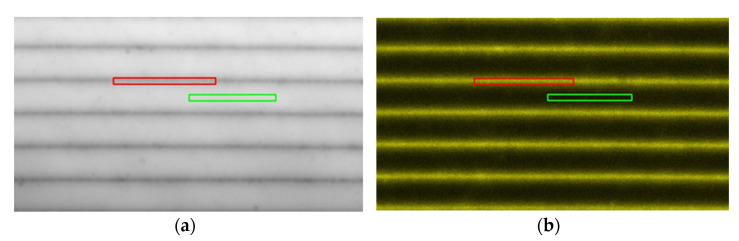
Transmitted light (**a**) and PL ((**b**), real color) images of a hologram with a spatial period of 2.5 μm obtained using an LSM 710 laser scanning microscope with a 405 nm laser. The rectangular regions in the bright and dark stripes represent those for which the local PL spectra were measured (see the spectra below).

Microscopic images of the holographic grating in transmitted light and PL obtained with a LSM 710 laser scanning microscope are presented in Figure 4.

Narrow dark stripes in transmitted light and bright stripes in PL separated by, respectively, broader light and dark stripes (Figure 4) apparently manifested the areas with a high QD concentration. According to the commonly accepted understanding of holographic recording in liquid photopolymer compositions with a neutral component [4,5], in the course of photopolymerization that occurs predominantly in the vicinity of the antinodes of the interference pattern, neutral nanoparticles (QDs) are expelled from antinodes to the nodes, while monomer molecules diffuse to the antinodes to become polymerized, thus resulting in alternating stripes of the polymer and the QDs.

**Figure 5:**
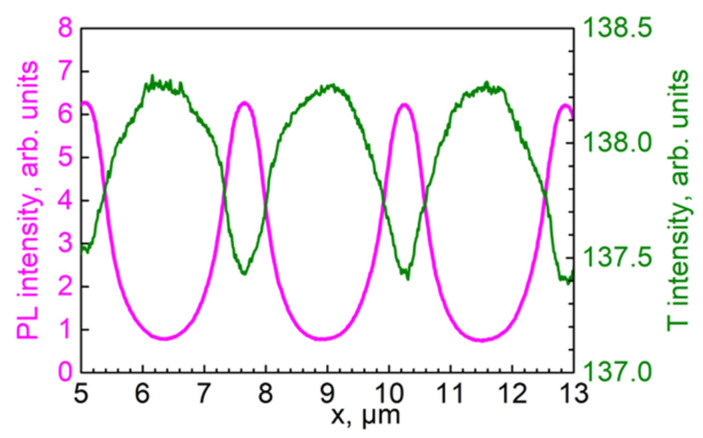
Intensity profiles of PL (magenta; excitation wavelength = 405 nm; acquisition range = 550–650 nm) and transmitted light (T, olive; 405 nm) along the holographic grating vector resulting from the data presented in Figure 4.

The profiles of PL and transmitted light intensity along the grating’s vector (across the stripes) obtained from the images of Figure 4 can be seen in Figure 5.

The profiles presented in Figure 5 noticeably deviated from the sinusoidal shape, thus revealing a nonlinear recording typical of holographic photopolymers [21]. Thus, the ratio of the amplitudes of the first four PL spatial harmonics in Figure 5 was 250:90:26:3. 

**Figure 6:**
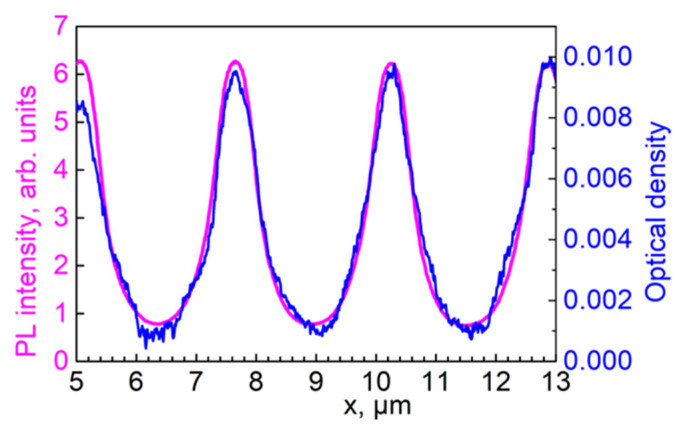
Merged PL intensity (magenta) and optical density (blue) profiles based on the data from Figure 5.

We supposed that the PL intensity of QDs in our samples depended linearly on the QDs’ concentration. The spatial dependence of the transmitted light’s intensity (Figure 5) correlated with an optical density profile. The PL and optical density profiles shown in Figure 6 almost coincided, and the maximum absorbance at 405 nm of about 0.01 is in fair agreement with the surface-average optical density of 0.005 measured with the spectrophotometer. However, a slight discrepancy between the profiles implied variations in the PL quantum yield, and this was proportional to the ratio of PL intensity to optical density, within 20%.

**Figure 7:**
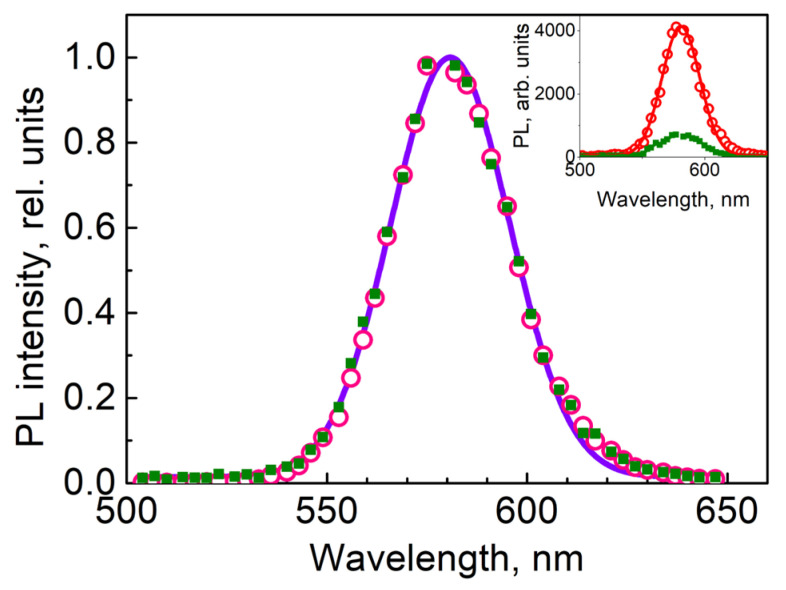
Local PL spectra of QDs in the regions of bright (open symbols) and dark (full symbols) stripes of the holographic structure with high and low concentrations of QDs, respectively (see the rectangular regions in Figure 4) normalized to the maximum values; the solid line represents the common Gaussian fit. Inset: the same PL spectra at the original intensity scale, with Gaussian fitting.

The local PL spectra of the QDs residing both in the bright and dark stripes of the grating measured with the LSM 710 confocal microscope proved to be identical in shape to the spectrum of those QDs in the liquid monomer, as Figure 7 shows, while the PL intensity in the light and dark stripes of the holographic grating differed by about one decimal order.

**Figure 8:**
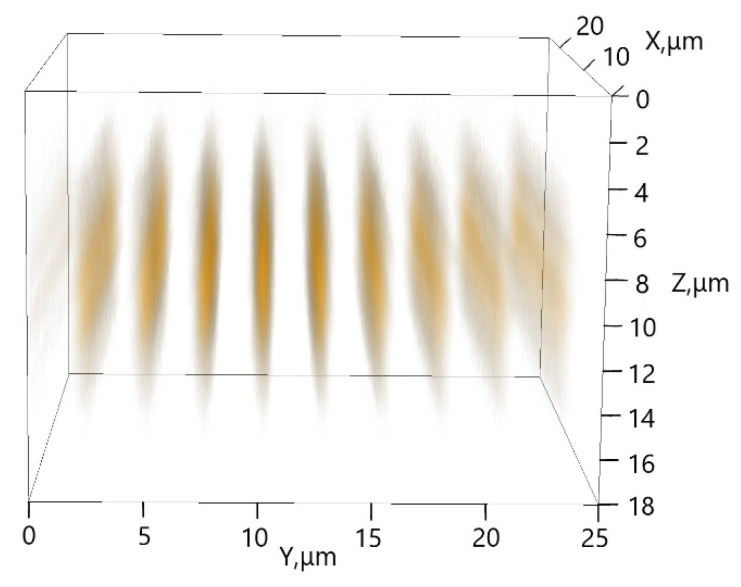
Three-dimensional PL image of a grating 10 µm thick with the QDs visualized using an LSM 710 laser scanning microscope. PL excitation wavelength, 405 nm.

The 3D image of the holographic grating (Figure 8) obtained using the LSM 710 confocal microscope by scanning along three coordinates with a 405 nm laser beam revealed no observable PL inhomogeneities that would represent microscale aggregates of QDs throughout the whole 10 µm thickness of the grating.

On the basis of (i) the maximum optical density at 405 nm of *D*_405 nm_ = 0.01 estimated from the absorbance profiles shown in Figure 6, (ii) the extinction coefficient $\varepsilon_{405\mathrm{nm}}=\varepsilon_{\mathrm{Peng}}D_{405\mathrm{nm}}/D_{\mathrm{exciton}}$= 4·10^4^ M^−1^ cm^−1^ that was proportional to $\varepsilon_{\mathrm{Peng}}$ = 1.25·10^5^ M^−1^ cm^−1^ determined for the exciton maximum using Peng’s formula [18] from the QD absorbance spectrum presented in Figure 2, and (iii) the grating thickness *l* = 10 ± 1 µm observed in Figure 8, the QD concentration in the maxima (antinodes) could be estimated using the Beer–Lambert–Bouguer law as $C=D_{405\mathrm{nm}}/\varepsilon_{405\mathrm{nm}}l$ = 5·10^−5^ M. This value corresponds to a distance between QDs of 30 nm, which is sufficiently large to avoid Förster resonance energy transfer (FRET) [22]. However, the average value cannot guarantee the uniform spatial distribution of QDs and does not exclude QD aggregation and FRET within QD aggregates. Evidence of its absence or presence can be provided by studies of PL kinetics aimed at looking for the signatures of energy transfer, as described in the next subsection.

### 3.5. Time-Resolved Laser Scanning Microscopy of a Holographic Grating

**Figure 9:**
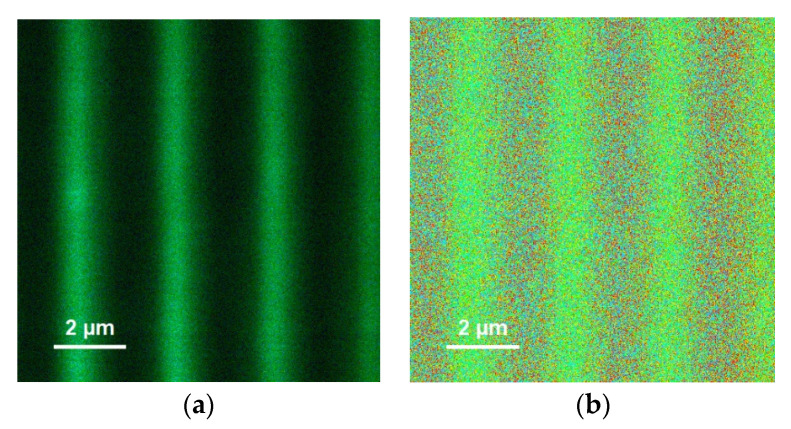
PL intensity image (**a**) and fluorescence lifetime image (**b**) of a grating with QDs. The excitation wavelength was 409 nm and the acquisition wavelength was 580 nm near the maximum of the QDs’ PL.

Nanoscale QD aggregates may manifest themselves via FRET, which would be revealed as the PL’s lifetime dependence on the QD concentration and acquisition wavelength (e.g., in [23]). A visual representation of the spatial lifetime distribution or FLIM (fluorescence lifetime imaging microscopy) pattern of the grating demonstrated a periodical structure apparently corresponding to the PL intensity view (Figure 9), hence implying the PL’s lifetime dependence on the QD concentration.

**Figure 10:**
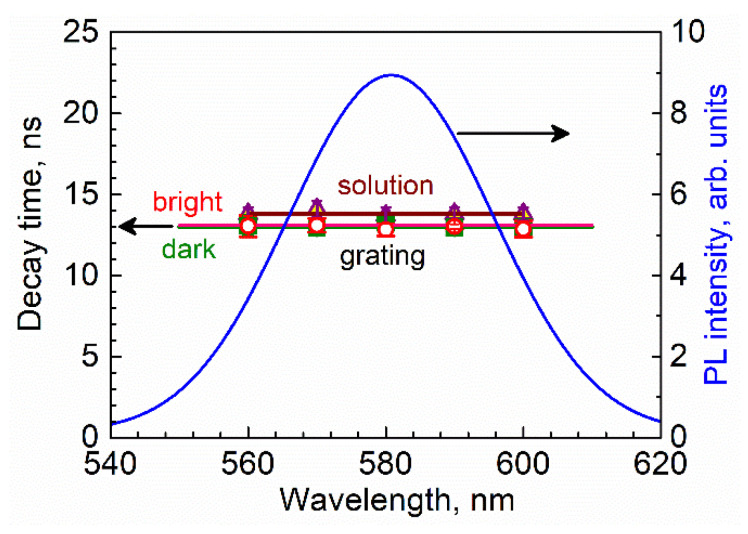
Average QD PL decay times in the bright (open symbols) and dark (full symbols) stripes of the grating and in a solution vs. the acquisition wavelength within the QDs’ PL band. The Gaussian representation of the PL spectrum is shown with blue line.

However, a comparison of the more thoroughly analyzed PL decay of QDs measured within their PL spectral band at the 10 nm spectral regions centered at 560, 570, 580, 590, and 600 nm demonstrated neither the spectral dependence of PL decay time nor the difference between QDs located in the dark and bright stripes of the grating, or in the liquid monomers, as Figure 10 shows. The set of data from the QD PL decay analysis is presented in Table A1, and the formulae used for the analysis are also included in Appendix B. The apparently “long” decay time initially observed in the dark stripes of the grating (Figure 9) are likely to be caused by the contribution of noise.

The absence of the significant wavelength dependence of the QDs’ PL decay times and their equal within the error bars and the values obtained for QDs located in the bright and dark stripes of the photopolymerized grating, as well as in the liquid monomer (Figure 10), clearly show the negligible efficiency of FRET between the QDs and hence the absence of QD aggregation at the nanoscale level.

## 4. Conclusions

To summarize, a luminescent grating with QDs periodically distributed in a polymer layer was formed, based on exposure to an interference pattern, photopolymerization, and photoinduced diffusion. In the formulation of the photosensitive composition, identical molecular species served both as a surface stabilizer for the QDs and a monomer to be photopolymerized, to achieve the natural affinity of QDs with the surrounding medium.

Using steady-state and time-resolved luminescence laser scanning microscopy, we proved the absence of both nano- and microscale QD aggregates in the grating.

Microscopic PL visualization of the periodic structure indicated the absence of micrometer-scale aggregates; the spectral and spatial independence of local PL decay proved the absence of resonant energy transfer between QDs, and hence the absence of even nanoscale aggregation. Thus, nanostructured luminescent gratings comprising monomer-coated QDs can be candidates for the role of hybrid diffraction–luminescent sensor elements.

## 5. Patents

Russian Patent RU 2752026 “Holographic photopolymerizable material” (published 22.07.2021, application 2020141309 of 15.12.2020) originated from part of the work reported in this study.

## Data Availability

Not applicable.

## Appendix A

Optical scheme used for holographic recording.

## Appendix B

**Table A1:** QD PL decay parameters in the monomer solution, and the bright and dark stripes of a grating: amplitudes, decay times of three exponential components, and the average decay time.

Acquisition Wavelength, nm	*A*_1_	*A*_2_	*A*_3_	*τ*_1_, ns	*τ*_2_, ns	*τ*_3_, ns	〈τ〉, ns
Solution							
560	0.74 ± 0.09	1.75 ± 0.08	0.08 ± 0.01	4.6 ± 0.5	15 ± 1	68 ± 5	13.8 ± 0.6
570	0.63 ± 0.0	1.79 ± 0.033	0.087 ± 0.006	3.7 ± 0.3	15 ± 1	62 ± 2	14.1 ± 0.6
580	0.74 ± 0.06	1.64 ± 0.04	0.066 ± 0.006	4.7 ± 0.4	15 ± 1	67 ± 3	13.7 ± 0.5
590	0.73 ± 0.04	1.75 ± 0.05	0.07 ± 0.01	4.1 ± 0.4	15 ± 1	67 ± 3	13.8 ± 0.6
600	0.76 ± 0.07	1.72 ± 0.06	0.10 ± 0.01	4.6 ± 0.5	15 ± 1	62 ± 4	13.8 ± 0.5
Bright stripes							
560	0.23 ± 0.02	0.36 ± 0.07	0.020 ± 0.005	4.4 ± 0.8	14.7 ± 0.6	72 ± 6	13.1 ± 0.7
570	0.45 ± 0.08	0.8 ± 0.1	0.05 ± 0.01	3.8 ± 0.5	14.3 ± 0.6	65 ± 3	13.5 ± 0.5
580	1.1 ± 0.1	0.7 ± 0.2	0.067 ± 0.005	4.2 ± 0.3	14 ± 1	64 ± 4	12.8 ± 0.4
590	1.1 ± 0.2	0.76 ± 0.09	0.06 ± 0.01	4.7 ± 0.3	14.3 ± 0.6	69 ± 3	13.0 ± 0.1
600	0.6 ± 0.1	0.7 ± 0.1	0.048 ± 0.006	5.4 ± 0.8	15 ± 1	68 ± 5	12.9 ± 0.5
Dark stripes							
560	0.11 ± 0.03	0.20 ± 0.04	0.013 ± 0.006	4 ± 1	15 ± 1	71 ± 15	13.1 ± 0.6
570	0.29 ± 0.06	0.37 ± 0.05	0.019 ± 0.004	4.7 ± 0.7	16 ± 1	79 ± 8	13.0 ± 0.4
580	0.35 ± 0.04	0.56 ± 0.08	0.031 ± 0.003	4.4 ± 0.1	14.3 ± 0.6	72 ± 3	13.3 ± 0.3
590	0.330 ± 0.005	0.49 ± 0.09	0.032 ± 0.006	4.6 ± 0.7	14.3 ± 0.6	66 ± 4	13.0 ± 0.4
600	0.25 ± 0.05	0.39 ± 0.06	0.023 ± 0.05	4.6 ± 0.3	14.7 ± 0.6	72 ± 5	12.9 ± 0.3

The QD PL decay was fitted by a 3-exponential function:

$$I(t)=I_{0}+A_{1}\exp\left(-\frac{t}{\tau_{1}}\right)+A_{2}\exp\left(-\frac{t}{\tau_{2}}\right)+A_{3}\exp\left(-\frac{t}{\tau_{3}}\right)$$

The average decay times were calculated by considering only the two shorter decay times typical of QDs, while the longest one with a smaller contribution, supposedly attributable to delayed PL, was neglected. The following formula for the average decay time [24] was used:

$$\langle \tau \rangle =\frac{A_{1}\tau_{1}^{2}+A_{2}\tau_{2}^{2}}{A_{1}\tau_{1}+A_{2}\tau_{2}}$$
